# Evolution of Premotor Cortical Excitability after Cathodal Inhibition of the Primary Motor Cortex: A Sham-Controlled Serial Navigated TMS Study

**DOI:** 10.1371/journal.pone.0057425

**Published:** 2013-02-21

**Authors:** Sein Schmidt, Robert Fleischmann, Rouven Bathe-Peters, Kerstin Irlbacher, Stephan A. Brandt

**Affiliations:** Department of Neurology, Charité-Universitätsmedizin Berlin, Berlin, Germany; Charité University Medicine Berlin, Germany

## Abstract

**Background:**

Premotor cortical regions (PMC) play an important role in the orchestration of motor function, yet their role in compensatory mechanisms in a disturbed motor system is largely unclear. Previous studies are consistent in describing pronounced anatomical and functional connectivity between the PMC and the primary motor cortex (M1). Lesion studies consistently show compensatory adaptive changes in PMC neural activity following an M1 lesion. Non-invasive brain modification of PMC neural activity has shown compensatory neurophysiological aftereffects in M1. These studies have contributed to our understanding of how M1 responds to changes in PMC neural activity. Yet, the way in which the PMC responds to artificial inhibition of M1 neural activity is unclear. Here we investigate the neurophysiological consequences in the PMC and the behavioral consequences for motor performance of stimulation mediated M1 inhibition by cathodal transcranial direct current stimulation (tDCS).

**Purpose:**

The primary goal was to determine how electrophysiological measures of PMC excitability change in order to compensate for inhibited M1 neural excitability and attenuated motor performance.

**Hypothesis:**

Cathodal inhibition of M1 excitability leads to a compensatory increase of ipsilateral PMC excitability.

**Methods:**

We enrolled 16 healthy participants in this randomized, double-blind, sham-controlled, crossover design study. All participants underwent navigated transcranial magnetic stimulation (nTMS) to identify PMC and M1 corticospinal projections as well as to evaluate electrophysiological measures of cortical, intracortical and interhemispheric excitability. Cortical M1 excitability was inhibited using cathodal tDCS. Finger-tapping speeds were used to examine motor function.

**Results:**

Cathodal tDCS successfully reduced M1 excitability and motor performance speed. PMC excitability was increased for longer and was the only significant predictor of motor performance.

**Conclusion:**

The PMC compensates for attenuated M1 excitability and contributes to motor performance maintenance.

## Introduction

The human motor system comprises distinct primary (M1) and secondary motor areas. Strong interconnections orchestrate interactions relevant to everyday activities [1], [2]. Artificial modulation or pathological alteration of any part can induce changes in network activity and underlying electrophysiological properties with consequences for proper motor functioning [3]–[6]. In general, functionally significant adaptations in premotor areas (PMC) after M1 lesions are well established in both animals [7] and humans [5], [7]–[10]. In macaque monkeys it has been shown that transient pharmacological PMC inhibition following functional recovery after ibotenic acid M1 lesions severely inhibited motor performance [11]. A similar effect can be observed in humans: inhibition of the ipsi- or contralesional dorsal PMC after M1 ischemic lesions inhibits motor performance [8], [12]. Furthermore, functional imaging studies confirm that behavorial compensation after M1 lesions depends strongly on the PMC activity [11], [13], [14].

Thus, several non-invasive brain stimulation studies have specifically investigated the relationship between PMC and M1 regions after single-pulse, inhibitory or facilitatory stimulation of the PMC to disclose the electrophysiological properties of interregional pathways mediating aforementioned effects [15]–[21]. The studies have consistently found strong connectivity between the PMC and M1 regions. The results strongly suggest the existence of a pathway targeting an interneuron network within M1 facilitating its corticospinal output. Other studies have established the behavioral consequences of M1 inhibitory stimulation for motor performance [4], [6], [14] and interhemispheric interactions between bilateral primary motor areas [19], [22]. Finally, lesion studies in animals and humans have consistently shown the significant role that the PMC plays in compensating for lost M1 functionality. In contrast, the compensatory changes in the PMC following the artificial inhibition of M1 neural activity and the behavioral consequences remain to be investigated. The structural basis for compensatory interactions can be found in (1) the vast array of interconnections between M1 and the PMC as well as in (2) corticospinal output neurons within the PMC [21].

Many aspects of the significance of the association between the PMC and M1 regions remain unresolved [18], [23], [24]. For example, Johansen-Berg et al. find that post-stroke motor performance depends on PMC activity in the contralesional hemisphere [25] while Fridman et al. provide evidence that ipsilesional PMC activation is beneficial and that contralesional regions make no substantial contribution [26]. Yet, Nelles et al. report that ipsilesional PMC activity can be either beneficial or disadvantageous [27]. A possible reason for these divergent findings could be that the amount of deficit plays a critical role in a time-dependent manner [5], [7], [12].

Swayne et al., based on lesion data obtained from stroke patients, proposed a multistage model of motor network compensation [23]. The model suggests that ipsilateral premotor function can only sufficiently compensate for lost M1 functionality in cases of mild impairment. Stepwise processes can recruit contralateral motor areas depending on the amount of deficit [5]. Similarly, a recent study also performed on stroke patients by Rehme et al. has shown how non-affected primary and secondary motor regions change their interaction patterns, i.e. facilitatory or inhibitory, over time [10]. This being said, evidence for time-dependency following non-invasive brain stimulation has also been found in a study on healthy test subjects by Lang et al. [28]. They investigated the temporal dynamics of tDCS induced net aftereffects in and between the bilateral primary motor cortices. They show that the aftereffects on interhemispheric inhibition and the intracortical excitability are time-dependent since local intracortical measures outlast changes in interhemispheric transmission [19]. Despite general similarities, clearly the underlying mechanisms must be assumed to be at least partially different to those seen in lesion studies since the timescale and intervention strength of tDCS does not allow for structural changes [29].

In summary, studies on patients clearly suggest there is an adaptation of the ipsilesional PMC to an M1 lesion that has significant behavioral consequences. However, it remains unclear whether the adaptation is beneficial or disadvantageous. Pathways with different impacts on M1 excitability have been identified in brain stimulation studies. Knowledge of these pathways is relevant to understanding the aforementioned M1-PMC interactions after an M1 lesion. However, the response of M1 and PMC pathways to systematically modified M1 excitability has not been studied. Moreover, none of these studies have related their electrophysiological findings to measures of motor function. Thus, our primary objective was to inhibit M1 neural activity and to study the effect this intervention has on PMC excitability, M1-PMC interaction, and motor performance in healthy test subjects. To address changes in intracortical circuitry and cortical excitability we focused on paired-pulse and input-output TMS protocols. To address the temporal dynamics, in line with a multistage hypothesis, we compared split-half results from a well-established 0 to 40 with a 40 to 80 minute ‘late-phase’ time window. Motor performance was measured by finger-tapping speed. We found that cathodal tDCS inhibition of M1 leads to enhanced PMC excitability associated with changes in motor performance.

## Methods

### Ethics approval

This study was approved by the Ethics Commission of the Charité Universititätsmedizin - Berlin and conformed to the *Declaration of Helsinki*. All participants provided written informed consent for the experimental procedure.

### Participants

We enrolled 16 participants, 15 right-handed, 1 left-handed (laterality index -90), 4 female, mean age 25.6±2.3 years, and took a detailed medical history to exclude neurological or psychiatric illness and the presence of implanted electronic devices or ferromagnetic metals. Handedness was confirmed by the Edinburgh handedness inventory.

### Design

All of the participants took part in three experimental sessions. All of the sessions took place at approximately the same time of day and the participants were asked to have had a sufficient night's rest before the experiments were conducted since the time spent awake has recently been shown to possibly affect susceptibility to non-invasive brain stimulation [30]. First, we performed navigated transcranial magnetic stimulation (nTMS) to determine the optimal stimulation site for M1 and PMC corticospinal projections in the dominant hemisphere. This was performed in an isolated session prior to the two tDCS stimulation sessions to avoid fatigue effects. The coordinates of the two stimulation targets were stored for use in following sessions. In the two subsequent stimulation sessions, sham and cathodal tDCS were applied over M1 individually in each subject. The order was randomized to avoid sequence effects. Both the participants and the investigator were unaware of the stimulation condition (double-blind design). The investigator was blinded by providing a numerical code which had to be entered to the stimulation software and was decoded into the stimulation type (Spike2, CED, Cambridge, UK). Additionally, the display of the stimulation device was covered. Immediately before and after tDCS, 20 TMS stimuli were applied at equal intensities (%MSO, i.e. percentage of maximum stimulator output) at 500 µV-MT (please refer to the section ‘*Corticospinal, intracortical and interhemispheric excitability*’ for a detailed definition of ‘500 µV-MT’) before tDCS to examine changes in cortical excitability induced by the stimulation.

**Figure 1:**
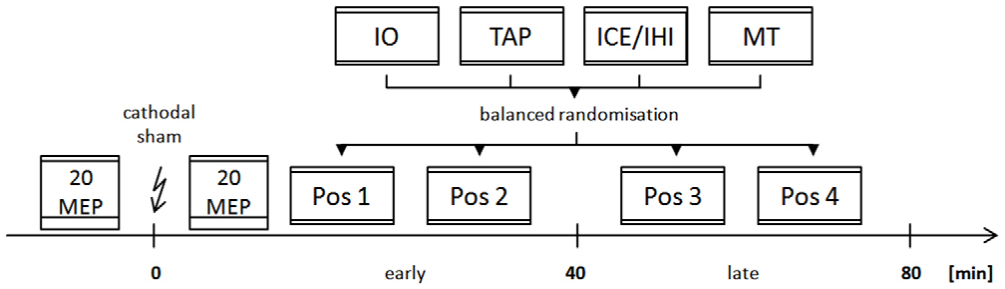
Diagram illustrating the randomization of parameters to be examined in one exemplary subject. Parameters of motor cortical excitability and function were assessed at two time intervals (early or late) following transcranial direct current stimulation (indicated by a flash symbol). Changes in measures recorded 0–40 minutes after discontinuation of the stimulation were considered to be due to early aftereffects. Changes recorded in the successive 40 minutes were considered to be due to late aftereffects. Aftereffects which occured in only one recording period were considered to be short-lasting; others were considered to be long-lasting. Immediately before and after tDCS, 20 stimuli were applied at a fixed intensity (see methods section for details) over M1 to examine tDCS-induced changes of corticospinal excitability. Afterwards, the five neurophysiological or functional parameters being evaluated were randomly assigned to one of four time slots in the overall 80-minute post-stimulation period. IO  =  input-output curve, TAP  =  finger-tapping, ICE  =  intracortical excitability (Short-interval intracortical inhibition (SICI) and intracortical facilitation (ICF), IHI  =  interhemispheric inhibition, MT  =  motor threshold; Pos1 – Pos4: Random position in time for each parameter.

Additionally, five measures of cortical excitability and motor function were each randomly assigned to one of four twenty-minute time slots distributed over an early (up to 40 minutes post-tDCS) and late (40–80 minutes post-tDCS) recording period. Two of these measures, interhemispheric inhibition and intracortical excitability (i.e. intracortical facilitation and inhibition), were assigned a common slot. The remaining three measures were input-output curves, motor thresholds, and finger-tapping speed (FT) (see Figure 1). The random order was kept constant in both tDCS conditions (sham versus verum) for each subject (i.e. stratified randomization). Overall, each electrophysiological and functional parameter was investigated on 8 occasions per time interval (i.e. early or late) and condition. We expected to capture all tDCS-induced aftereffects within 80 minutes [31]. Previous work indicates that the duration of tDCS-induced aftereffects varies among different measures of intracortical and interhemispheric excitability [19], [22]. The relative change across these five parameters in the post stimulation period was compared between stimulation conditions in a within subject design (repeated measures analysis of variance (ANOVA)).

### Finger tapping

The participant's dominant hand rested on a custom-built copper board that enabled index finger tapping with minimal effort and no mechanical resistance. Participants were instructed to start with and maintain a maximum tapping frequency for 30 seconds for the duration of the trial. Trials were started with a visual cue.

### Navigated transcranial magnetic stimulation (nTMS)

Individual structural MRI (3D-MPRAGE, matrix 256×256, 180 sagittal slices, voxel size 1 mm^3^, on a GE 3 Tesla scanner) were acquired. The participant's head was tracked by an infrared-based stereotactic system and brought into co-registration with the MRI using a triangular system of anatomical landmarks (bilateral tragus and nasion) as well as a subsequent 9-point surface registration.

We used the eXimia system (Nexstim Ltd, Helsinki, Finland) to calculate the strength, location, and direction of the stimulating electric field in the cortical tissue which was derived from a dynamic spherical model. This information was adjusted in real time, and individual head size, shape, and physical parameters of stimulation were taken into account [32]. TMS pulses were delivered through an eXimia TMS stimulator connected to a focal monophasic figure-of-eight coil (70 mm outer diameter; Nexstim Ltd, Helsinki, Finland). Figure-of-eight coils affect a cortical area of about 1.7 cm^2^ [33] or smaller [34], [35] with a rapid decay of effect size to the margin [36] and with a precision comparable to that of intraoperative direct cortical stimulation [37] at a predefined peeling depth of between 20 and 25 mm. EMG samples from the eXimia system (sampling rate 3000 Hz) were obtained with the participants seated in a comfortable reclining chair and instructed to relax and keep their eyes open. Surface EMG electrodes (Neuroline 700, Ambu, Ballerup, Denmark) were attached to the abductor pollicis brevis (APB) and biceps brachii (BB) muscles of the arm contralateral to the stimulated hemisphere. Trains of similar stimuli were applied with interstimulus intervals (ISI) randomized between 1 and 3 seconds.

### Primary and premotor cortical mapping

During the first session, individual bilateral M1 and PMC regions were mapped for an abductor pollicis brevis (APB) stimulation hotspot defined by the maximal motor evoked potential (MEP) response site with minimal suprathreshold TMS intensity. Accurate mapping was facilitated by using a more focal monophasic stimulator instead of the more commonly-used and stronger biphasic type [38] with stimulation perpendicular to the underlying gyral projections.

External landmarks were identified for PMC regions and subsequently the PMC location was mapped for direct corticospinal output. The eXimia system provides the estimated maximum electric field strength induced under the coil (EF_max_) and at any remote site (EF_remote_) [39]. It employs a spherical model of the cortical surface, which has been validated for cortical targets in stimulation studies as well as preoperative identification of M1 [37], [40]. After PMC hotspot identification, 20 minimal suprathreshold stimuli were applied at an intensity eliciting MEPs at an average amplitude of 200 µV. This confirmed the reliable elicitation of corticospinal volleys. The concomitant EF_remote_ in M1 was estimated. Subsequently, 20 stimuli were applied with this EF_remote_ intensity and orientation directly over the M1 hotspot. If no MEPs were elicited then the MEPs that had been elicited by PMC stimulation were assumed to originate in the PMC and not to be due to distant co-stimulation of M1. This approach is a modified version of that successfully used by Teitti et al. [21]. They argued that if the EF_remote_ over M1 during frontal or premotor stimulation was below a level sufficient to elicit an MEP from M1 (i.e. below RMT), then the MEP must have originated from corticospinal projections other than those located in M1. Spangenberg et al. have reproduced this finding in brain tumor patients [41]. We built upon these findings by including control stimulation over M1 to prove that the assumption of a non-M1 origin is correct.

### Corticospinal, intracortical and interhemispheric excitability

The MEP amplitude was defined by peak-to-peak measurements of belly-tendon surface recordings from the muscles contralateral to the dominant hemisphere with a background activity below a maximum of 20 µV. Motor thresholds were defined within a 95% confidence interval by an efficient maximum-likelihood algorithm [42] and are given as a percentage of the maximum stimulator output (%MSO) required to elicit an average MEP response of 50 µV (i.e. resting motor threshold, RMT), or 500 µV (termed ‘500 µV-MT’) or 1 mV (termed ‘1 mV-MT’). Input-output curves were assessed by randomly applying ten stimuli at an intensity of 110%, 120%, 130% and 140% of the individual's RMT [22]. The associated assessment of cortical excitability at multiple stimulation strengths can provide explicit information about the target area [43], and importantly about corticospinal excitability per se as a measure of the common final descending pathway of all neuronal subsets. Care was taken to measure a reliable RMT, which was estimated per subject, measure and time slot individually. Interhemispheric inhibition (IHI) was measured bi-directionally using two TMS coils placed over the APB hotspots of the primary motor cortices of each hemisphere (Coil 1: Nexstim, Helsinki, Finland; Coil 2: Magstim 200, Magstim, UK). The navigation system was also used to place the second coil over the respective hotspot with high spatial precision. The conditioning stimulus (CS) trigger preceded the test stimulus (TS) trigger by 10 ms [44]. Intertrial intervals were set at random lengths of between 3–5 seconds. The stimulus intensity was set to 1 mV-MT for both the conditioning as well as the test stimulus. 20 stimuli were applied either with a CS preceding the TS or with the TS only, in a randomized order to avoid sequence effects. Short-interval intracortical inhibition (SICI) and intracortical facilitation (ICF) were tested with paired-pulses at interstimulus intervals (ISI) of 3 and 10 ms respectively [45]. CS were applied at 80% RMT and TS at the individual 1 mV-MT intensity. A total number of 20 stimuli were applied for each ISI. Subsequently, 20 stimuli with the TS only were taken as baseline.

### Transcranial direct current stimulation (tDCS)

A bipolar constant current stimulator (DS5, Digitimer, CED, Cambridge, UK) was used to apply tDCS. The waveform was controlled by a digital-analog converter (Power1401 DAC and Spike2 software, both CED, Cambridge, UK). The center of the stimulation electrode was placed over the APB hotspot of the primary motor cortex of the dominant hemisphere using co-registered hotspots in individual MRI by the eXimia stereotactic system (Nexstim, Helsinki, Finland). The electrode's surface area was adapted to avoid effective stimulation of the adjacent PMC, to prevent high scalp current densities and to elicit an aftereffect in the stimulated M1 of about one hour [4], [46]. A 16 cm^2^ (6.4×2.5 cm) cathodal strip electrode of less than half the size of the 35 cm^2^ electrodes used in early tDCS studies [22] was used and placed with its long axis parallel to the central gyrus to further diminish spread to the rostrally located PMC. The reference electrode, with a size of 35 cm^2^, was placed over the supraorbital region of the opposite hemisphere to disperse the current to a non-effective level [4]. The electrodes were soaked in 0.9% sodium chloride solution and attached to the participant's head with adhesive tape.

A current of −0.7 mA with a current density of −0.04 mA/cm^2^ was used [4]. Estimating the current density on the surface has been shown to be complex. Results vary depending on spherical or cylindrical models. For optimal predictive power, a geometric head model derived from actual MRI data should be used [47]. The cathodal tDCS consisted of ramping the current to −0.7 mA over 5 seconds, keeping it at this level for 9 minutes 50 seconds, and ramping it to 0 mA in another 5 seconds to avoid the unpleasant sensations that occur when rapidly switching the current on or off. Sham tDCS was also started with a 5-second ramping phase to −0.7 mA. This level of current was kept constant for 30 seconds (compared to 10 minutes verum stimulation) to give the participant a tingling sensation. The current was subsequently reduced to 0 mA over another 5 seconds and then kept at this level until a total of 10 minutes sham-stimulation was completed.

### Data evaluation and statistics

MATLAB (Matlab®, Mathworks, Gatwick, USA) was used for signal processing and statistical testing. The strength of inhibition or facilitation in paired-pulse trials was quantified as a percent change normalized to the baseline. In accordance with established estimations of CSE [48], [49], 20 MEP response input-output curve examinations were z-transformed to account for inter-individual MEP variability. For presentation, the normalized data were scaled to a real dimension in mV.

Electromyographic recordings were controlled online and post-hoc for muscle pre-innervation exceeding 20 µV since pre-innervation is known to be associated with increased MEP size unrelated to intervention and possibly masking aftereffects [50]. This was done both by visual inspection of EMG traces during stimulation as well as post-hoc thresholding and regression analysis prior to data preprocessing. MEP onset latencies were automatically determined by the Nexstim software with an algorithm that utilizes the first deflection from the baseline of smoothed MEP traces.

A one-way analysis of variance (ANOVA) was calculated to compare mean MEP amplitudes before and after tDCS. A repeated measures ANOVA was calculated to test the null hypothesis that additionally recorded parameters between stimulation conditions (i.e. cathodal and sham) or MEP latencies between regions (i.e. M1 and PMC) did not differ (Table 1). Time was introduced as a two-level main factor for all tests because early and late aftereffects were expected to be different. TMS intensity was added as a four-level main factor for input-output curve testing as high and low-threshold neurons have been found to be affected differently by tDCS [4]. To determine if inhibitory or facilitatory circuits were affected, the interstimulus interval was included as a two-level main factor in paired-pulse trials [45]. If the null hypothesis was rejected at a 5% significance level, we performed a Tukey's honestly significant difference (HSD) post-hoc test as an alpha-error cumulative-corrected multiple comparison procedure.

**Table 1:** Summary of experimental results.

Interregional		*df*	F	p	
*MEP Latency*					
	Region	1	4.51	0.0380	*
	Muscle	1	0.58	0.4490	
	Region x muscle	1	0.61	0.4395	
Functional					
*Maximum tapping frequency*					
	Intervention	1	1.05	0.3109	
	Time	1	0.47	0.4981	
	Intervention x Time	1	6.39	0.0152	*
Primary motor cortex					
*Corticospinal Excitability*					
Intervention		1	18.63	0.0000	*
*Resting motor threshold*					
	Intervention	1	1.96	0.1669	
	Time	1	0.19	0.6608	
	Intervention x Time	1	6.61	0.0129	*
*Input-output curve*					
	Intervention	1	0.50	0.4799	
	Time	1	0.04	0.8368	
	TMS intensity	3	286.33	0.0000	*
	Intervention x Time	1	0.30	0.5865	
	Intervention x TMS intensity	3	3.84	0.0094	*
	Time x TMS intensity	3	0.38	0.7702	
	Intervention x TMS intensity x Time	3	3.04	0.0280	*
*Paired pulse stimulation*					
	Intervention	1	2.07	0.2489	
	Time	1	8.43	0.0039	*
	ISI	1	72.78	0.0000	*
	Intervention x Time	1	0.74	0.0615	
	Intervention x ISI	1	1.50	0.1230	
	Time x ISI	1	10.02	0.0017	*
	Intervention x ISI x Time	1	5.68	0.0030	*
*Interhemispheric inhibition (non-dominant to dominant hemisphere)*					
	Intervention	1	7.01	0.0102	*
	Time	1	1.15	0.2873	
	Intervention x Time	1	0.12	0.7258	
*Interhemispheric inhibition (dominant to non-dominant hemisphere)*					
	Intervention	1	0.43	0.5179	
	Time	1	0.14	0.7133	
	Intervention x Time	1	0.46	0.5026	
Premotor cortex					
*Resting motor threshold*					
	Intervention	1	0.89	0.3551	
	Time	1	1.32	0.2604	
	Intervention x Time	1	5.59	0.0255	*
*Input-output curve*					
	Intervention	1	22.13	0.0000	*
	Time	1	0.11	0.7397	
	TMS intensity	3	208.58	0.0000	*
	Intervention x Time	1	0.18	0.6699	
	Intervention x TMS intensity	3	3.19	0.0246	*
	Time x TMS intensity	3	1.57	0.1975	
	Intervention x TMS intensity x Time	3	1.61	0.1872	

ANOVA results have been categorized with respect to whether data was compared between brain *region*s (i.e. primary motor cortex (M1) and dorsal premotor cortex (PMC)) or *intervention*s (i.e. cathodal and sham stimulation) and whether they examine functional performance or electrophysiological properties. The test of MEP latency contains the two-level factor *muscle* for forearm and hand muscles. The two-level factor *time* distinguishes between early(<40 min) and late (>40 min) period aftereffects. The four-level factor *TMS intensity* refers to 10% increments of the individual resting motor threshold (RMT) used as stimulation intensities to assess input-output curves. Paired-pulse stimulation sequences contain the two-level factor ISI reflecting SICI and ICF protocols. Please refer to the methods section for further details. * p<0.05.

We used a stepwise regression model to identify the electrophysiological parameters that were significant predictors of change in functional data (i.e. finger-tapping speed). To identify regional effects, measures of corticospinal and intracortical excitability from both M1 as well as from the PMC for verum conditions were included in the model.

Mean data is always given ± its standard error. Mean differences between groups are given with the upper and lower margin of their 95% confidence interval (95% CI) in brackets. Bootstrapping was used to estimate the accuracy of the mean optimal electric field location and stimulus orientation of M1 and PMC hotspots within a 95% CI.

## Results

### Premotor mapping

PMC hotspots were successfully identified in all participants. The population average location of PMC hotspots was 17.09 mm rostral (95% CI: 14.94–18.99 mm) and 14.64 mm medial (95% CI: 12.52–16.73 mm) to the M1 hotspot on the cortical surface. Optimal coil orientation for M1 and PMC stimulation differed significantly (4.44°, 95% CI: 3.44–5.38°). Latencies of MEPs elicited by PMC stimulation (21.76 ms±0.82) were comparable (−0.88 ms, 95% CI: −1.43–3.19 ms) to those following M1 stimulation (22.64 ms±0.82) (F_(1,15)_ = 0.58, p = 0.45). Results did not differ between different muscle groups with respect to the target regions (F_(1,15)_ = 0.61, p = 0.44) (Table 1). However, MEP response latencies recorded proximally from the biceps were shorter (F_(1,15)_ = 4.51, p = 0.04; difference: −1.78 ms, 95% CI: −1.49 – −5.05 ms).

### Motor function

**Figure 2:**
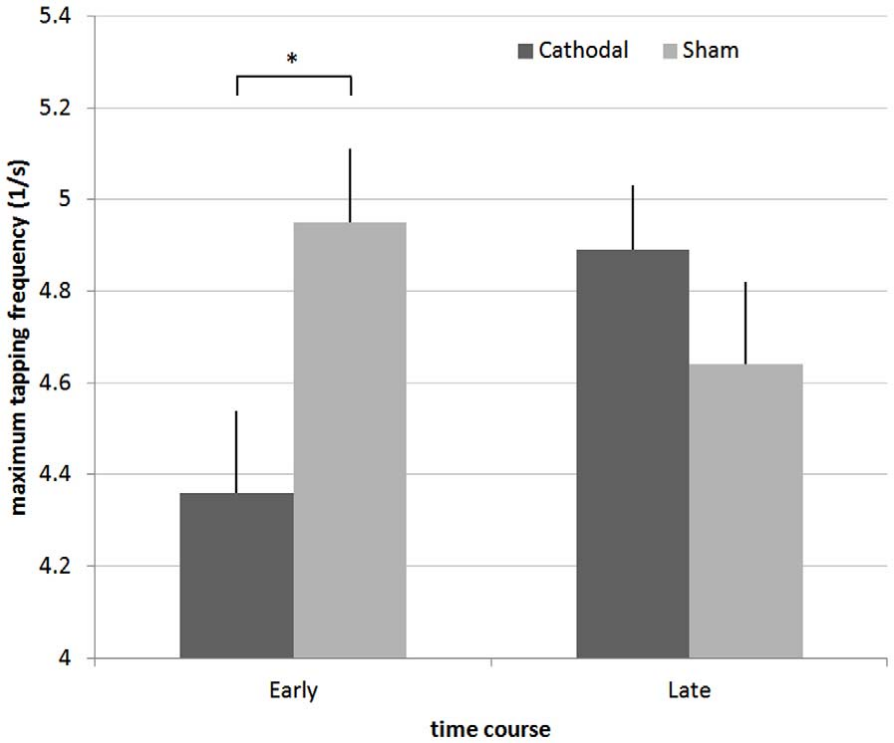
Maximum finger-tapping frequency reached in 30 seconds before and after 40 minutes (see Figure 1). Error bars represent the standard error of the mean. Finger-tapping speed is significantly slowed directly after cathodal stimulation as compared to sham stimulation or the late period. * p<0.05

Cathodal tDCS had a significant time-dependent effect on maximum finger-tapping frequency (F_(1,15)_ = 6.39, p = 0.02). Post-hoc testing revealed that participants were significantly slower in the early period following cathodal tDCS (11.91%, 95% CI: 2.63–1.21%) than when having received sham tDCS (p<0.05) (Figure 2). There was no difference between the groups in the late period.

### Electrophysiological parameters

#### Primary motor cortex

**Figure 3:**
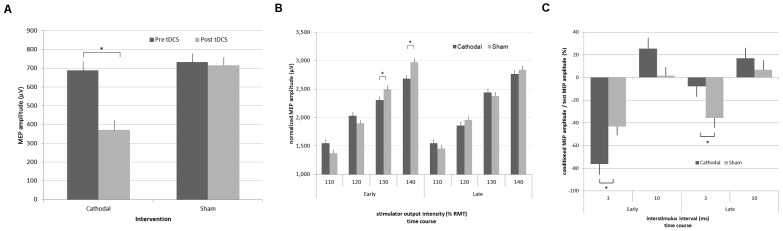
Results and comparison of electrophysiological responses to either cathodal or sham stimulation in the primary motor area. A) Cortical excitability estimates. MEP average amplitude in the early and later period. In contrast to sham tDCS, cathodal tDCS significantly diminished mean MEP amplitudes by about 50%. B) Input-output curves. MEP average amplitudes at 110% through 140% RMT. In contrast to sham tDCS, cathodal tDCS significantly diminished mean MEP amplitudes at stimulation strengths of 130% and 140% RMT. C) Short-interval intracortical inhibition (SICI) and intracortical facilitation (ICF). Average amplitudes of the test MEP at 2 and 5 ms ISI. SICI is enhanced in the early and significantly reduced in the late post-stimulation period after cathodal stimulation. ICF is not significantly affected by the cathodal stimulation. The time periods in all figures correspond to the definition of time intervals in Figure 1. Error bars represent the standard error of the mean. * p<0.05

Cathodal stimulation resulted in a decreased mean MEP amplitude by about 50% (688±50 µV vs. 370±54 µV; F_(1,15)_ = 18.63, p<0.05). In contrast, MEP amplitudes after sham stimulation were similar to those obtained before stimulation (732±46 µV vs. 717±44 µV; F_(1,15)_ = 0.06, p = 0.8) (Figure 3a).

Stimulation significantly influenced resting motor thresholds (RMT; F_(1,15)_ = 6.61, p = 0.013) between groups in the post-stimulation period when time was considered. Post-hoc testing revealed that RMT was 9.18% (95% CI: 0.04 – 18.33%) higher in the cathodal stimulation condition compared to sham stimulation in the early period (p<0.05). There was no late period aftereffect.

Input-output curve evaluation provided further evidence for a time-dependent inhibitory aftereffect when TMS stimulation intensity was considered (F_(3,15)_ = 3.04, p = 0.03). Post-hoc testing revealed that MEP amplitudes were 7.73% (95% CI: 0.32–15.15%) and 9.79% (95% CI: 2.74-12.73%) lower at TMS intensities of 130% and 140% RMT in the early post-tDCS period when preceded by cathodal stimulation instead of sham (p<0.05). In the late period, in line with effects on RMT, MEP amplitudes were unchanged (Figure 3b).

Paired-pulse examinations revealed a clear relationship between intervention type, time and ISI (F_(1,15)_ = 5.68, p = 0.003). Post-hoc testing showed that in the early period, SICI following cathodal tDCS was significantly stronger at 32.95% (95% CI: 8.91 – 56.99%) than SICI following sham tDCS. Second level interactions suggest that paired-pulse stimulation might also be dependent on time, independent of intervention type (F_(1,15)_ = 10.02, p = 0.002). Post-hoc tests show that SICI was stronger in the early period than in the late period, independent of the tDCS type (−59.77%±6.11 vs.−21.74%±6.41, p<0.05).

Unlike all other measures of cortical excitability, SICI displayed a sign change in the late period, being 28.05% (95% CI: 2.82–53.28%) weaker in the cathodal condition. In contrast, ICF was unaffected by either time or stimulation (Figure 3c).

We found that the IHI in the dominant interventional hemisphere differed significantly between tDCS conditions (F_(1,15)_ = 7.01, p = 0.01). Post-hoc testing revealed a mean inhibition of corticospinal excitability of 45.64%±9.38 in the cathodal vs. 15.97%±8.48 in the sham condition in the early post-stimulation period (95%CI of mean difference: −4.4% – −54.93%) in the ipsilateral interventional hemisphere. There was no significant difference in the late period or in the contralateral hemisphere.

#### Premotor cortex

**Figure 4:**
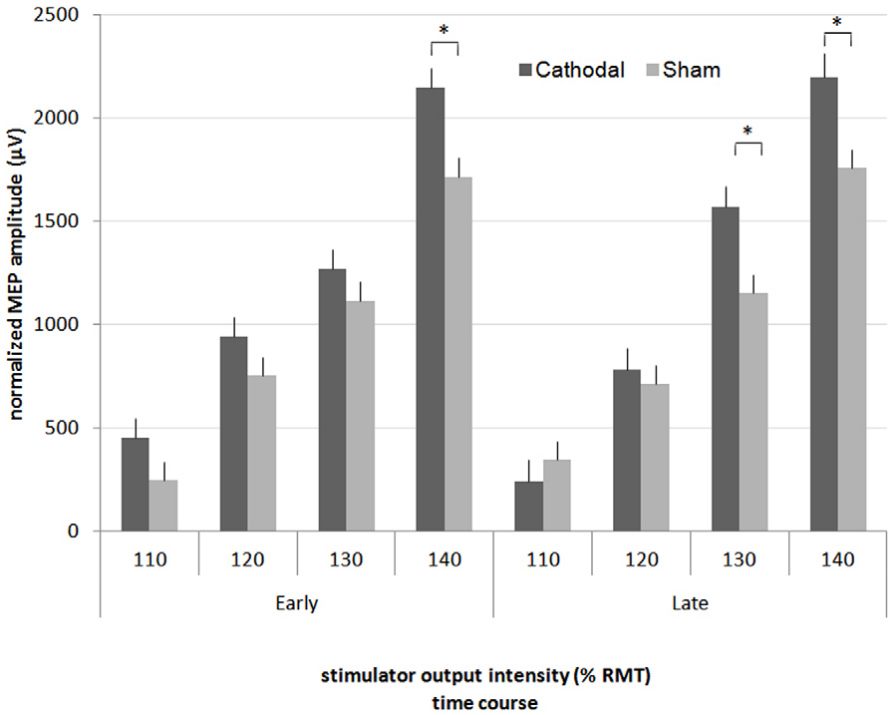
Input-output curves over PMC. MEP average amplitudes defined by stimulation at 110% through 140% RMT. In contrast to sham tDCS, cathodal tDCS significantly enhanced cortical excitability. Significant results were found at 130% (late period) and 140% (both periods) RMT. Error bars represent the standard error of the mean. * p<0.05

Corticospinal excitability (CSE) evaluation revealed post interventional evidence for enhanced premotor CSE. MEP responses were significantly different for the individual TMS intensities (F_(3,15)_ = 3.19, p = 0.03). Post-hoc testing revealed that MEP amplitudes in the early period were 25.42% (95% CI: 9.92 – 40.92%) higher in the cathodal condition than in the sham condition (p<0.05) when assessed at 140% RMT. In the late period, MEP amplitudes were higher in the cathodal condition when assessed at 140% as well as 130% RMT (35.6%, 95% CI: 12.39 – 58.82% and 25.19%, 95% CI: 9.07 – 41.32%). RMT changes were intervention and time-dependent (F_(1,15)_ = 5.59, p = 0.03). Post-hoc testing revealed a late-onset increase in premotor RMT of 14.15% (95% CI: 1.98 – 26.91%) in comparison to sham tDCS (p<0.05) (Figure 4).

### Regression analysis

Stepwise regression analysis showed a significant positive correlation between MEP elicited at 130% RMT in the premotor area and finger-tapping performance (RMSE = 0.99, R^2^ = 0.51, p = 0.046). Other results from primary or premotor areas did not show a significant relationship.

## Discussion

The main findings of this study are that (1) ipsilateral PMC excitability increased after cathodal-tDCS-induced inhibition of M1, (2) PMC excitability increase outlasted established M1 excitability decrease and (3) PMC excitability at 130% RMT was the only predictor of faster finger-tapping speed. These results show that the PMC is affected by M1 cathodal inhibition with neurophysiological as well as behavioral consequences.

### The site of cortical inhibition

To target M1 but not PMC with tDCS we adopted the methods suggested by Nitsche et al. [4] and Miranda et al. [47] and used a stimulation electrode with a smaller surface area (16 cm^2^ vs. conventional 35 cm^2^), a rectangular instead of a quadratic shape, and reduced total current (−0.7 mA vs. conventional −1.0 mA). Although previous brain stimulation studies with tDCS have successfully used larger electrodes to differentiate PMC from M1 effects (e.g. 35 cm^2^ leads to opposite metabolic effects in ipsilateral M1 and dorsal PMC [14]), we believe that a more focal stimulation will prove to be preferable. Conversely, whereas 35 cm^2^ electrodes have successfully differentiated effects in M1 and PMC, 9 cm^2^ electrodes were recently shown to be inefficient [51]. Thus, the success of stimulation with a 16 cm^2^ surface area must be measured by the strength of inhibition induced in M1. The findings of this study show that a 16 cm^2^ tDCS electrode can induce increased SICI, lateralized IHI, increased RMT and reduced corticospinal excitability in accordance with well-established cathodal tDCS effects [4], [19]. This confirms that the use of a smaller electrode was successful. Unlike Nitsche et al. we did not find a reduced ICF. However, this is not a novel finding and can be explained by more focal stimulation [15]. Similar to Lang et al. we found IHI to only be changed in the interventional hemisphere and not in the contralateral hemisphere [19]. However, Lang et al. found IHI to be decreased after cathodal tDCS, unlike the results in our study [19]. This might also be attributed to the use of smaller stimulation electrodes (16 cm^2^ vs. 35 cm^2^). In summary, a smaller electrode than is typically used to differentiate M1 and PMC effects can effectively inhibit M1 electrophysiological and behavioral functions.

### Sites of neurophysiologic assessment

To localize the PMC we followed previous studies that derived the location of the PMC from well-established external measures and anatomical landmarks (e.g. 2 cm rostral to M1) (see for example, [1], [8], [17], [41], [52]). For further validation we utilized neurophysiological parameters provided by navigated brain stimulation, as described recently [21], [53]. We found the localization of the PMC by navigated stimulation to be successful. The distances between PMC and M1 hotspots were comparable to established anatomical landmarks [54]–[56]. Thus, navigated brain stimulation can localize and help distinguish intracortical M1 from PMC target sites.

Premotor areas have been shown to be amenable to non-invasive brain stimulation methods by either single-pulse TMS [8], [21], [52] or plasticity-inducing stimulation protocols such as tDCS [15] and rTMS [16], [20], [57]. Building on multiple previous studies that investigate PMC stimulation and differentiate M1 effects [15]–[17], [52], [57], and as described previously [21], [53], we retrieved volts per meter estimates to add additional confirmation that PMC stimulation did not induce co-stimulation of the (on average 2.25 cm) distant M1 site. Conversely, one might contend that the eXimia system's spherical model estimates validated for a cortical surface are not adequate for the depth of e.g. the central sulcus [36]. More explicitly, in this case, PMC stimulation might induce undetected distant co-activation of muscles in M1 and elicit MEPs either directly or indirectly via volume conduction in the APB hotspot itself or from muscles with lower thresholds than the APB [58]. This would be more critical at higher stimulation strengths, i.e. in this study for the input-output curves. We found similar, i.e. a non-significant trend to faster response latencies [21], [53], reduced M1 and enhanced PMC corticospinal excitability. This study, like previous studies, cannot exclude the possibility that the future non-spherical estimation of deep currents along the anterior wall of the central sulcus might provide evidence for higher current densities in M1. Conversely, spurious co-activation cannot explain the success of previous studies in selective modification of PMC functions by tDCS or TMS [8], [12], [15], [20], [52] nor this study's findings of similar response latencies (for PMC and M1 stimulation) and decreased M1 yet enhanced PMC excitability after cathodal stimulation. Additionally, for direct corticospinal tract activation by stimulation of the subcortical white matter, the MEP response latencies would have been remarkably shorter [59] and amplitudes not affected by cathodal tDCS [60]. Conversely, the response latencies would have been remarkably longer in the case of activation via a PMC-M1 corticocortical pathway. None of these cases apply to the results found in this study. The results of this study, found by taking measurements over a well-established distant premotor site with focal nTMS and tDCS stimulation, match best with the notion of indirect ‘posterior-anterior’ stimulation of high-density projections originating in the PMC, which have been demonstrated convincingly in animal tracer studies [29].

### Aftereffects found in PMC after cathodal tDCS over M1

This is the first study to investigate the electrophysiological properties of the PMC following cathodal M1 stimulation. However, several studies have assessed M1 response to PMC interventions [15], [16], [20], [51], [52], [57]. These studies find both inhibitory and facilitatory pathways between the PMC and M1 [15], [17], [20], [57] possibly related to gaba'ergic circuits [15], [16], [61] and dependent on both stimulation strength and frequency [20].

We find that artificially reduced M1 activity leads to reduced SICI in M1 and heightened PMC activity. Boros and colleagues found that artificially heightened PMC activity (anodal tDCS in their study) leads to suppression of SICI (i.e. gaba'ergic inhibition) in M1 [15]. Activity in the PMC was not assessed, so that a more direct comparison is not possible. The effect on SICI in the present study is not apparent in the early post-stimulation period, most likely because gaba'ergic interneuronal activity (intrinsic activity) is masked by the direct inhibitory tDCS aftereffects (extrinsic input) on gaba'ergic interneurons [4]. In summary, the gaba'ergic results are similar, suggesting, at least in part, the utilization of some similar pathways. Concerning effects on corticospinal excitability, Gerschlager et al. show that PMC inhibition leads to reduced and Rizzo et al. show that heightened PMC neural activity leads to heightened M1 excitability [16], [57]. In line with these findings, we find enhanced (normalization of reduced) M1 excitability during enhancement of PMC excitability. Conversely, it is understood that M1 and PMC can share both facilitatory and inhibitory pathways relevant to the shaping and selection of movements [20]. Thus, the results of the present study could suggest that the facilitatory pathways are predominantly responsible for the compensation of attenuated movements. Yet the alternative explanation, enhanced inhibition of inhibitory pathways, is also plausible and the differentiation of these two pathway types remains unresolved by the present study.

Attenuated electrophysiological and behavioral findings in M1 are all well-established for cathodal tDCS [22]; e.g. with significant effects at 130% and 140% but not 110% RMT in input-output curves as well as similar effects on intracortical and interhemispheric circuits. Furthermore, we find clearly enhanced PMC excitability that is present both directly after cathodal tDCS and in a later period after M1 excitability has returned to normal. Interestingly, significant cortical excitability changes at 130% RMT are only present in this late period. Moreover, it is also the only predictor of motor performance. This selective effect is likely related to modification of the recruitment characteristics of corticospinal neurons [62], due to the ‘time-dependent’ and possibly NMDA-receptor-dependent modulation of different cortical systems [22]. A limitation to this study is that navigation software still cannot provide conclusive evidence against unspecific distant activation of low-threshold M1 neurons located e.g. at the depth of the precentral gyrus. This could have masked a possible association of motor performance with stronger stimulation strengths.

The finding of a secondary effect on the PMC after M1 inhibition is novel but not surprising as strong M1 PMC connectivity and compensatory mechanisms after M1 lesions are well established in both animals (for review see [7]) and patients [5], [8], [12], [13], [61], [63]. Further late period effects were also found for RMT and SICI, which, although not predictive of motor performance, suggest the existence of concurrent changes in PMC and M1 intracortical circuitry [22]. The specific finding of a predictive association between cortical excitability at 130% RMT and motor performance deserves further investigation. In summary, cathodal tDCS over M1 in healthy test subjects induces changes in the ipsilateral PMC cortical excitability as assessed by TMS that are associated with motor performance.

### A comparison with results from lesion studies

Presently, there is no non-invasive brain stimulation study in humans that has investigated how PMC responds to cathodal inhibition of M1. Conversely, as discussed above, studies in animals and humans clearly suggest that the PMC will respond strongly to a lesion-based impairment of primary motor function. Additionally, since tDCS has been successfully used in the recovery maximization of motor function in stroke patients through motor network modification, it seems tDCS partly interacts with and shares mechanisms of plasticity that occur in these patients. Thus, our results deserve a brief comparison. The results of this study suggest that future non-invasive brain stimulation studies might expect PMC excitability to be directly related to compensatory electrophysiological changes in M1 as well as motor performance. This is in line with previous evidence suggesting electrophysiological changes can precede and drive structural changes [64]; that reduced M1 excitability and prolonged reaction times in M1 impaired patients are associated with compensatory, possibly partially vicarious PMC modifications [7], [8], [12]; and that increased PMC metabolic activity has been associated with a better outcome in stroke patients [10], [65]. In contrast, increased PMC activity following M1 lesions has also been found to have a negative influence on motor performance [8], [61]. Additionally, the role of ipsi- versus contralateral PMC remains unresolved [8], [66]. These findings can possibly be explained by a direct association with the amount, extent, location and temporal dynamics of functional impairment and recovery processes in the central motor system [5], [7], [12], [13], [61]. The discussion of this study's results in the context of lesion studies is confined by the fact that the present study investigates healthy subjects after cathodal induction of an LTD-like effect that cannot elicit any structural changes and at best a transient functional perturbance [4], [31]. Conversely, this study shows that transient inhibition of the primary motor cortex has direct consequences for ipsi-interventional premotor neural assemblies and motor performance.

### Conclusions

The findings of this study confirm that the attenuation of ipsilateral primary motor cortex function in healthy test subjects leads to longer-lasting changes in the ipsilateral premotor region. These premotor changes can be predictive of faster finger-tapping speed and are possibly related to gaba'ergic mechanisms.
